# Can Sleep Hypoxic Measures Predict Long-Term Adherence to Positive Airway Pressure Treatments?

**DOI:** 10.3390/clockssleep8040061

**Published:** 2026-09-28

**Authors:** Andrea Portacci, Sara Quaranta, Carla Santomasi, Valentina Di Lecce, Vitaliano Nicola Quaranta, Monica Amendolara, Ilaria Dei Lazzaretti, Laura Anna Sara Cuccaro, Massimo Casparrini, Sebastiano Spierto, Vito Picerno, Cristina Derobertis, Alfredo Vozza, Silvano Dragonieri, Giovanna Elisiana Carpagnano

**Affiliations:** 1 Institute of Respiratory Disease, Department of Translational Biomedicine and Neuroscience, University “Aldo Moro”, 70124 Bari, Italy; a.portacci01@gmail.com (A.P.); sara.quaranta.02@gmail.com (S.Q.); carlasantomasi@gmail.com (C.S.); valentina.dilecce89@gmail.com (V.D.L.); vitalianonicola.40@gmail.com (V.N.Q.); m.amendolara2@studenti.uniba.it (M.A.); ilariadeilazzaretti@gmail.com (I.D.L.); cuccarolaura@gmail.com (L.A.S.C.); maxadhar@gmail.com (M.C.); spierto.sebastiano@gmail.com (S.S.); vito.picerno.97@gmail.com (V.P.); cri.derobertis@gmail.com (C.D.); elisiana.carpagnano@uniba.it (G.E.C.); 2 Department of Precision and Regenerative Medicine and Ionian Area, University of Bari “Aldo Moro”, 70124 Bari, Italy; alfredovozza@live.it

**Keywords:** TST90, CPAP, adherence, hypoxia, burden, sleep fragmentation

## Abstract

Obstructive sleep apnea (OSA) is a prevalent sleep-related breathing disorder characterized by recurrent upper airway collapse. Continuous positive airway pressure (CPAP) is the gold standard treatment but is burdened by low adherence rates. Nighttime hypoxia may alter sleep structure and OSA symptoms, potentially affecting PAP treatment adherence. This study aims to evaluate whether nocturnal hypoxia measures could predict long-term PAP adherence. We conducted a real-life, retrospective study including 207 OSA patients. Clinical data were collected on symptoms, comorbidities, home sleep apnea test (HSAT) metrics, prescribed therapy and objective adherence data via in-built device software. Nocturnal respiratory failure (NRF) was defined as a total sleep time with SpO_2_ < 90% (TST90) ≥ 30%. Among the cohort, 119 patients met the criteria for NRF. These patients exhibited higher BMI, more severe OSA and a higher prevalence of COPD compared to those without NRF. At follow-up, patients with NRF demonstrated significantly better treatment adherence and more frequently reported improvement in several daytime and nighttime symptoms. Time-to-event analysis and adjusted multivariable regressions showed a higher probability of good long-term PAP adherence in patients with TST90 ≥ 30%. These findings suggest that prolonged nocturnal hypoxemia may identify a subgroup of patients more likely to maintain long-term PAP treatment.

## 1. Introduction

Obstructive sleep apnea syndrome (OSA) is the most common respiratory disorder, resulting from complete or partial collapse of the upper airway during sleep and accompanied by persistent or increased respiratory effort aimed at maintaining sufficient airflow [1]. Several pathological traits have been recognized, such as structural susceptibility, a low arousal threshold that causes frequent awakenings, reduced activity of the pharyngeal dilator muscles and an unstable breathing control system, also referred to as elevated loop gain [2]. Regardless of the underlying pathophysiological factor, systemic consequences of this disturbance include hypoxia, hypoxemia, hypercapnia, elevated sympathetic activity and chronic inflammation, accompanied by endothelial disfunction and cellular apoptosis [3].

The current definition of obstructive sleep apnea (OSA) relies mainly on the apnea-hypopnea index (AHI), defined as the total number of apnea and hypopnea events per hour of sleep [4]. However, AHI does not include several important pathophysiological information related to OSA such as apnea-hypopnea length or hypoxia severity. From this perspective, intermittent and continuous hypoxia have been associated with increased cardiovascular events and mortality [5,6], whereas AHI and oxygen desaturation index (number of SpO_2_ desaturations ≥ 3% per hour of sleep, ODI)—could be less reliable indicators [7,8].

With increasing recognition of the heterogeneity of OSA, there is a growing shift toward treatment strategies tailored to specific phenotypes. While novel treatment options—including pharmacological interventions targeting individual pathophysiological traits—are emerging [9,10], continuous positive airway pressure (CPAP) remains the gold standard therapy [11]. However, adherence to CPAP therapy remains suboptimal in many cases due to several factors including sociodemographic variables, disorder severity, psychological distress and social influences [12,13]. For this reason, identifying predictive features associated with poor CPAP adherence could help clinicians develop targeted therapeutic strategies for patients at higher risk of treatment rejection.

Among nocturnal oximetry parameters, TST90, defined as the percentage of total sleep time spent with SpO_2_ below 90%, reflects the duration of nocturnal hypoxemia and may vary considerably among patients with similar AHI values [14]. A TST90 ≥ 30% has been previously used as an operational threshold to identify nocturnal respiratory failure (NRF), reflecting prolonged nocturnal hypoxemia [15,16]. Moreover, in a previous study, patients with more severe OSAS based on both AHI and TST90 were more likely to remain adherent to CPAP over time, suggesting that TST90 may help predict long-term adherence [17]. However, few studies have investigated the predictive value of nocturnal hypoxia metrics in relation to PAP therapy adherence. Therefore, our study aimed to investigate the value of sleep oximetry parameters in predicting long-term PAP adherence.

## 2. Results

### 2.1. Baseline Features

**Figure 1:**
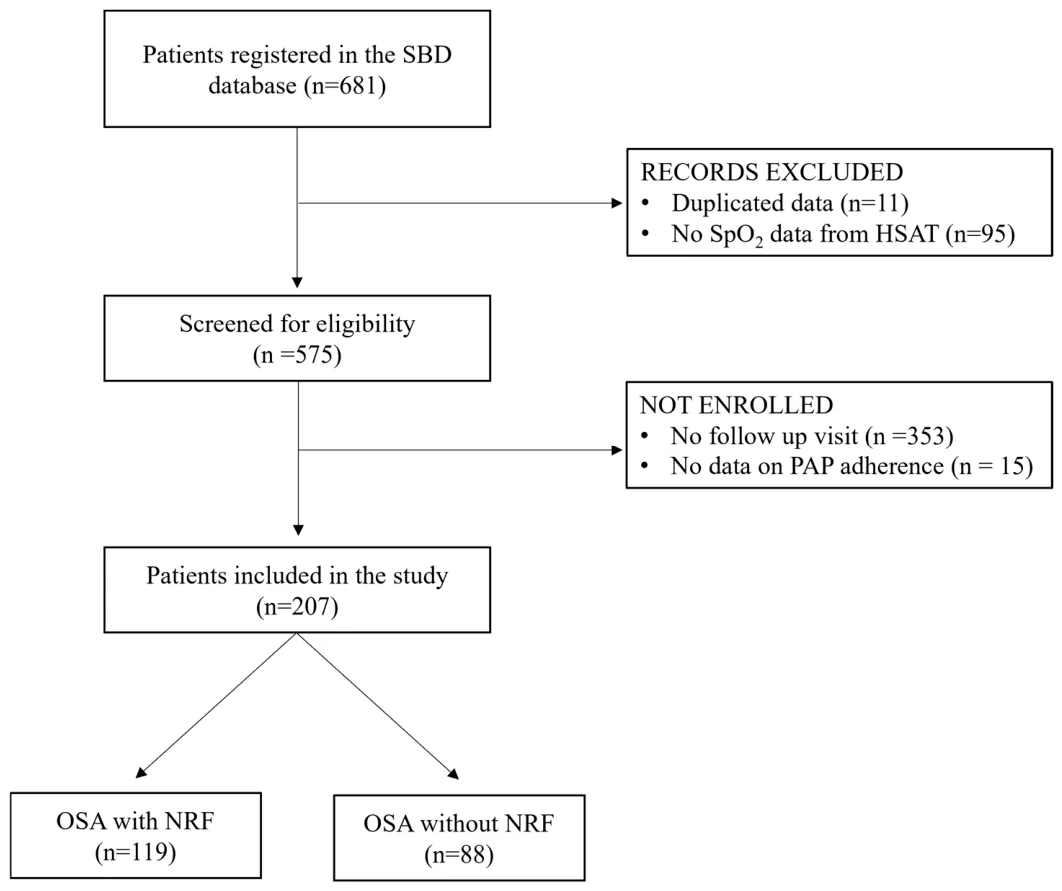
Flow chart showing the enrollment process for the overall population. Abbreviations: SBD, sleep breathing disorder; HSAT, home sleep apnea testing; PAP, positive airway pressure; OSA, obstructive sleep apnea; NRF, nocturnal respiratory failure.

After applying the exclusion criteria, 207 patients were included in the final analysis. Of these, 119 were diagnosed as having OSA with NRF, while 88 had a diagnosis of OSA without NRF (Figure 1).

The analysis of baseline features demonstrated that patients diagnosed with OSA with NRF had a higher BMI and larger neck circumference compared to those with OSA without NRF (*p* < 0.0001). We found no significant differences in reported daytime and nighttime symptoms as well as comparable ESS scores between the two populations (Table 1). Among comorbidities, the prevalence of COPD was significantly higher in the OSA with NRF group (21% vs. 6.9%, *p* = 0.005).

After performing HSAT, patients with NRF had worse OSA severity, with 76.5% of patients having an AHI ≥ 30 events/hour compared to the 42% of the group without NRF (*p* < 0.0001). As expected, patients with TST90 ≥ 30% had worse nocturnal hypoxic metrics (Table 2). Therapeutic strategies also differed in the two populations. Patients with OSA were more often treated with APAP (21.6% vs. 0.8%, *p* < 0.0001), whereas those with OSA and NRF had higher chances of being treated with BPAP therapy (11.8% vs. 1.1%, *p* = 0.003) and/or LTOT (8.4% vs. 1.1%, *p* = 0.03). No significant differences were found in the type of interface used between the two groups. However, median therapeutic CPAP pressure was significantly higher in the OSA with NRF group compared to the OSA without NRF group (11.7 cmH_2_O vs. 10 cmH_2_O, *p* < 0.0001).

### 2.2. Follow-Up Visit and Treatment Adherence

At follow-up visit, patients with OSA with NRF were more likely to adhere to the prescribed PAP treatment (69.7% vs. 52.3%, *p* = 0.01), with a median device use of 5.3 (3.4–6.2) hours vs 4.0 (1.3–5.5) hours (*p* = 0.002). Notably, the time from treatment prescription to the follow-up visit was shorter in patients with OSA and TST90 ≥ 30% than in those without NRF (271 vs. 513 days, *p* < 0.0001).

Patients with NRF experienced greater improvements in both daytime (sleepiness, insomnia, concentration disorders) and nighttime (snoring, nocturia) symptoms (Supplemental Table S1). Among the causes of discomfort related to PAP therapy, the most commonly reported were airway mucosal dryness, interface issues and unintentional air leaks, with no significant differences observed between the two groups (Table 3).

### 2.3. Predictors of Treatment Adherence

**Figure 2:**
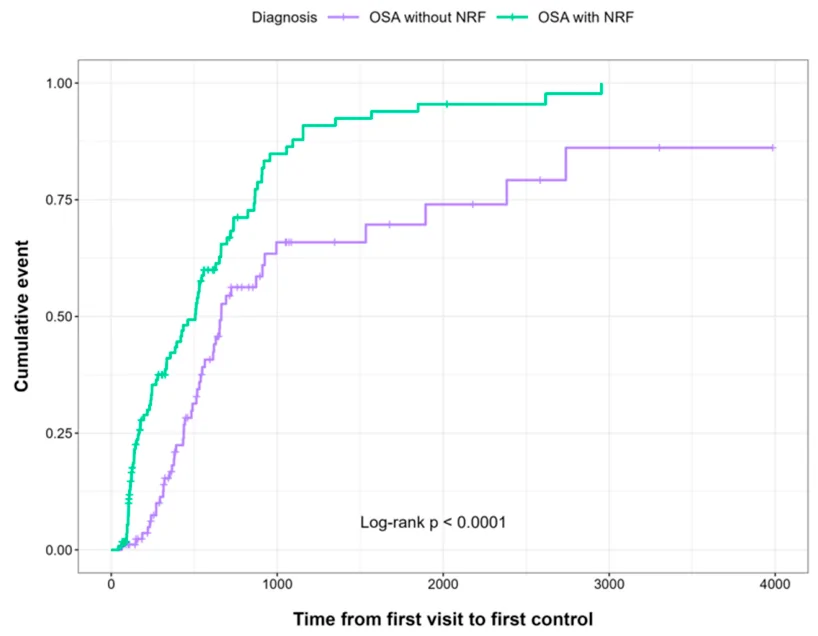
Kaplan-Meier curve for PAP treatment adherence according to the presence of nocturnal respiratory failure (NRF). Abbreviations: OSA, obstructive sleep apnea; NRF, nocturnal respiratory failure.

Using Kaplan-Meier curves with the log-rank test (Table 4), we found that patients with TST90 ≥ 30% had higher odds of maintaining a good adherence to the prescribed PAP therapy until along follow up (Figure 2, χ^2^ = 16.1, *p* < 0.0001). The result was similar even when considering the median TST90 value (34.9%) as cut-off for the analysis (χ^2^ = 9.4, *p* = 0.002). Among the other nocturnal hypoxia metrics, the mean SpO_2_ and the nadir SpO_2_ showed sufficient predictive power to identify patients with a higher likelihood of good PAP adherence in the OSA with NRF cohort (*p* = 0.01 and *p* = 0.02, respectively).

Further analysis using the LASSO Cox proportional hazard model found NRF to be a predictor for PAP treatment adherence (Table 5, AUC = 0.6, *p* = 0.02). The LASSO logistic regression model also confirmed these results, showing statistically significant predictive performance for these parameters (AUC 0.66, *p* = 0.006). When the median TST90 value (34.9%) was used as the cut-off, TST90 was retained in both the Cox and logistic LASSO models (Supplementary Table S2; AUC = 0.59, *p* = 0.02 and AUC = 0.66, *p* < 0.0001, respectively).

To ensure the robustness of our results, we performed two sensitivity analyses considering nadir SpO_2_ and mean SpO_2_ as variables for NRF definition. When using the median value of nadir SpO_2_ as cut-off (76%, Supplemental Table S3), we found good predictive power in both LASSO Cox (AUC = 0.61, *p* = 0.03) and logistic regression (AUC = 0.66, *p* < 0.0001) models. Differently, mean SpO_2_ was significantly associated with PAP adherence in the LASSO Cox regression model but not using the logistic regression analysis (Supplemental Table S4).

In the additional conventional multivariable logistic regression analysis, NRF remained independently associated with good PAP adherence (adjusted OR 2.52, 95% CI 1.24–5.13; *p* = 0.01). TST90 above its median value showed a non-significant trend toward greater adherence (adjusted OR 1.85, 95% CI 0.93–3.68; *p* = 0.08), whereas nadir SpO_2_ above its median value showed a non-significant trend toward lower adherence (adjusted OR 0.54, 95% CI 0.27–1.07; *p* = 0.08). The remaining metrics were not independently associated with the outcome (Supplemental Table S5).

## 3. Discussion

Our study demonstrated that, among the nocturnal hypoxia measures evaluated, NRF was the only variable independently associated with sufficient PAP adherence after adjustment for clinical characteristics, follow-up duration and treatment-related variables. In contrast, TST90 and nadir SpO_2_ dichotomized at their median values showed only non-significant trends, while the remaining hypoxia metrics were not independently associated with adherence. To the best of our knowledge, few published studies specifically addressed the role of several nighttime hypoxia measures as predictors of future treatment adherence. Campos-Rodriguez and collaborators highlighted the role of AHI-related TST90 to foreshadow CPAP adherence over a 4-year follow-up in patients without significant daytime sleepiness [17]. Similar results were found in patients using automatic PAP devices, with TST90 being the most valuable predictor of long-term treatment adherence [18]. Differently, Kohler et al. demonstrated that patients with lower baseline ODI were at higher risk of poor long term CPAP adherence [19].

In our study, ODI, TST85 and TST80 were not associated with PAP adherence over follow-up. Among the nocturnal hypoxia metrics evaluated, only NRF remained independently associated with sufficient adherence, while TST90 and nadir SpO_2_ showed borderline, non-significant associations. These findings may reflect a greater perceived disease burden and a more evident symptomatic benefit after PAP initiation, which could reinforce patients’ motivation to continue treatment. Supporting this hypothesis, patients with OSA and TST90 ≥ 30% reported greater improvements in daytime sleepiness (*p* = 0.009), insomnia (*p* = 0.01), memory loss (*p* = 0.07), concentration disorders (*p* = 0.02), snoring (*p* < 0.0001) and nocturia (*p* = 0.02) after treatment start (Supplemental Table S1). Notably, many of these and other daytime and nighttime symptoms improved or were completely resolved with consistent treatment, suggesting that the clinical improvement could have motivated patients in their PAP device use over time. Another important aspect could be related to the perception of severity disease. Patients’ perception of OSA severity is closely dependent on HSAT parameters such as AHI and TST90 [20]. In our cohort, patients diagnosed with OSA with NRF showed more severe OSA and nocturnal hypoxemia, which may have increased their awareness of disease burden and reinforced their motivation to adhere to treatment. However, these mechanisms were not directly assessed in our study and should be considered as speculative.

Finally, nighttime hypoxia could change sleep architecture, worsening sleep quality and influencing treatment adherence. In healthy subjects, acute exposure to hypoxia reduces TST, N3 and rapid eye movement (REM) sleep phases, increasing N1 stage length, arousal index and wakefulness time after sleep onset (WASO) [21]. However, chronic hypoxia effects on sleep architecture are less well known. Salvaggio et al., in a study investigating the changes in periodic breathing at high altitude, reported a sustained reduction in deep sleep stages which was only partially attenuated by a four-weeks adaptation period [22]. These results have been confirmed by Morrison and colleagues, who reported a significant decrease in slow-wave sleep along with a pronounced breathing instability after continuous exposure to hypoxia [23]. From a clinical standpoint, it may be hypothesized that correction of nocturnal hypoxia with regular PAP use could improve sleep architecture, contributing to long-term adherence. However, because HSAT does not assess sleep stages, this potential mechanism remains purely theoretical.

In our study, hypoxic burden showed only a non-significant association with PAP adherence in the Kaplan–Meier analysis and was not independently associated with the outcome in the conventional multivariable logistic regression. Although recent evidence has confirmed a strong association between HB severity, cardiovascular risk, and positive response to CPAP therapy [8,19], HB primarily reflects the intermittent nature of hypoxic burden, offering limited insight into the effects of sustained hypoxia. In contrast, TST90 captures both the intermittent and continuous components of nocturnal hypoxia, which may explain its stronger association with CPAP adherence compared to However, HB data were available for only 103 of the 207 patients, which may have reduced the statistical power of this analysis. Therefore, the lack of an independent association between HB and PAP adherence should not be considered definitive and requires further investigation in larger cohorts.

Although NRF was independently associated with adherence, its discriminatory performance was modest (AUC = 0.60 in the Cox analysis and 0.66 in the logistic LASSO model, respectively) and it should not be considered a standalone clinical predictor. AHI-based OSA severity and baseline sleepiness were not associated with adherence in the time-to-event analysis, although no formal comparison between separate prediction models was performed. Since TST90 is routinely available from HSAT, its potential clinical role should be considered complementary to a broader clinical assessment, and its predictive value requires prospective validation.

Our study has several limitations. First, the retrospective design and limited sample size may have reduced the statistical power of our findings. The analyses based on median cut-offs were exploratory and may have been affected by the loss of information and statistical power associated with dichotomization. Additionally, the use of HSAT rather than polysomnography prevented the assessment of sleep architecture, so the proposed relationship between nocturnal hypoxia, changes in sleep stages and PAP adherence should be considered as entirely speculative. Another limitation is the difference in follow-up duration between patients with and without NRF. Patients with NRF had a shorter follow-up and less time for adherence to decline, which may partly explain their higher observed adherence. Although follow-up duration was included as a covariate in the multivariable models, this adjustment may not have fully accounted for differences in follow-up timing, particularly because follow-up visits were not scheduled according to a standardized protocol. Residual confounding related to follow-up timing therefore cannot be excluded. PAP treatment was also not standardized, as treatment modality was selected according to individual clinical requirements. Although PAP modality, LTOT and interface type were included in the multivariable models, residual confounding related to treatment selection remains possible. Finally, as stated previously, HB data were available for only 103 patients, potentially limiting the statistical power of our results.

## 4. Methods and Materials

### 4.1. Study Design

We performed a retrospective, single-center study enrolling patients with OSA from January 2020 to June 2024 from the Sleep Disorders Clinic at Bari University Hospital. At baseline visit, we collected electronic medical records data on patients past medical history, comorbidities, anthropometric measures (body mass index (BMI), neck circumference) daytime and nighttime symptoms, and Epworth sleepiness scale (ESS) scores. Then, OSA diagnosis was confirmed using home sleep apnea testing (HSAT) following American Association of Sleep Medicine (AASM) recommendations [4]. Patients with AHI ≥ 15 or ≥ 5 in the presence of relevant respiratory symptoms or comorbidities underwent in-lab or home PAP titration according to clinical judgment. The prescribed treatment modality included fixed CPAP, auto-adjusting positive airway pressure (APAP) or bilevel positive airway pressure (BPAP), and was selected according to individual clinical requirements.

Follow-up visits included an assessment of symptom evolution after treatment initiation. For each daytime and nighttime symptom, patients were asked to compare its status with the previous visit, and the response was classified as worsened, unchanged, improved or absent. Follow-up visits also included changes in body weight and ESS, sleep duration, PAP adherence assessed through the device’s built-in time counter, and reasons for treatment-related discomfort. High treatment adherence was defined in the presence of a mean device use from baseline to the last follow-up visit ≥ 4 h/night. Patients without at least one follow-up visit or without retrievable data on PAP adherence were excluded from the analysis. Moreover, we also excluded patients with an unreliable SpO_2_ signal due to artifacts on HSAT.

This study was approved by the institutional ethics committee of our center (ethics committee number: 6750) and was conducted in accordance with the Declaration of Helsinki (1975) and Good Clinical Practice standards.

### 4.2. Hypoxic Burden Calculation

To evaluate the potential role of intermittent hypoxia in predicting PAP adherence, we calculated the hypoxic burden (HB) referring to previously published papers [24,25]. The first step involved identifying all desaturations that exceed a 2% threshold compared to the baseline value. For each desaturation event, a segment of SpO_2_ signals was extracted from 100 s before, until 100 s after, the nadir (Time Zero), and the signals were aligned relative to Time Zero. Next, a search window was defined for each desaturation, extending from the pre-maxima (the peak closest to Time Zero but at least 5 s away) to the post-maxima (the first peak after Time Zero). For each identified interval, the area under the baseline—defined as the starting SpO_2_ level of each desaturation—was calculated using numerical integration methods. Finally, all calculated areas were summed and normalized to the total sleep time to obtain HB, expressed in %min/h (Supplemental Figure S1). Overlapping desaturations were defined as consecutive events in which a second fall in SpO_2_ occurred before complete recovery from the preceding event, resulting in intersecting pre-maximum-to-post-maximum intervals. Only events with distinct, non-overlapping intervals were included, to prevent the same portion of the SpO_2_ signal from being integrated twice and overestimating HB. The pre-maxima were defined as the closest peak before Time Zero, but at least 5 s away, to improve the identification of independent events. We also implemented a control to ensure that the duration between pre-maxima and post-maxima was between 5 and 150 s. Moreover, the normalization of the areas was performed considering the total available recording time for each subject.

### 4.3. Statistical Analysis

Continuous variables distribution was assessed with the Shapiro-Wilk test. We expressed variables with a normal distribution as means ± standard deviations (SD) and those with a non-normal distribution as medians and interquartile ranges (IQR). Student’s t test and the Mann-Whitney test were used for comparisons. Categorical variables were expressed as percentages and compared using Fisher’s exact test or the chi-square test.

To evaluate the probability of high treatment adherence according to clinical and HSAT variables, we performed Kaplan-Meier time-to-event analysis with log-rank tests. For this analysis, we defined as nocturnal respiratory failure (NRF) the presence of a total sleep time with SpO_2_ < 90% (TST90) ≥ 30%, as previously reported in the literature [16,26]. All the hypoxic metrics were also evaluated as binary variables using their median values as cut-offs (>median vs. ≤median).

Then, we tested the predictive power of Kaplan-Meier curves using two multivariable least absolute shrinkage and selection operator (LASSO) regression models. LASSO regressions select variables significantly associated with the outcome using a penalty (λ) that excludes non-relevant covariates [27]. The first model was a LASSO Cox multivariate time-to-event regression, while the second one was a LASSO logistic regression.

As a sensitivity analysis, we performed separate multivariable logistic regression models to evaluate the association between each nocturnal hypoxia metric and good PAP adherence. NRF was analyzed according to the predefined TST90 ≥ 30% threshold, whereas the other hypoxic metrics were dichotomized using their median values. We also repeated the multivariable logistic regression with TST90 entered as a continuous variable and the OR expressed per 10-percentage-point increase. All these models were adjusted for age, sex, BMI, AHI, chronic obstructive pulmonary disease (COPD) diagnosis, the total number of comorbidities, follow-up duration, PAP modality (CPAP/APAP vs. BPAP), long-term oxygen therapy (LTOT) and interface type (nasal/nasal pillows vs. oronasal).

For each model we also calculated the receiver operating characteristics (ROC) curves with their areas under the curves (AUC), to ensure a proper robustness of the analysis.

All statistical analyses were performed using SPSS 26.0 (SPSS Inc., Chicago, IL, USA) and R software (version 4.0.2, R Foundation), considering a *p*-value < 0.05 as statistically significant.

## 5. Conclusions

In conclusion, NRF was independently associated with good long-term PAP adherence, whereas the other nocturnal hypoxia metrics were not related with the outcome. These findings suggest that prolonged nocturnal hypoxemia may identify a subgroup of patients more likely to maintain PAP treatment, although its use as a standalone clinical predictor remains to be established. Prospective longitudinal studies are needed to confirm these observations and clarify the potential underlying mechanisms.

## Figures and Tables

**Table 1:** Baseline anthropometric and clinical features according to NRF diagnosis.

	OSA w119.	OSA Without NRF (*n* = 88)	*p* Value
Age (Years, Mean, SD)	62 ± 11	62 ± 9	0.44
Male/Female (%)	76.5/23.5	64.8/35.2	0.09
Weight (Kg, Median, IQR)	100 [88–115]	86 [78–95]	<0.0001
BMI (Kg/m^2^, Median, IQR)	34 [31–39]	30 [28–33.2]	<0.0001
Neck circumference (cm, Median, IQR)	44 [41–46]	41 [39–43]	<0.0001
ESS (Median, IQR)	6 [3–10]	6 [3–10]	0.95
Daytime Symptoms (%, *n*)			
Sleepiness	56.3 (67)	55.7 (49)	0.99
Fatigue	42.9 (51)	38.6 (34)	0.57
Headache	19.3 (23)	13.6 (12)	0.35
Insomnia	56.3 (67)	56.8 (50)	0.99
Memory Loss	35.3 (42)	47.7 (42)	0.09
Concentration disorders	26.9 (32)	35.2 (31)	0.22
Mood disorders	38.7 (46)	38.6 (34)	0.99
Nighttime symptoms (%, *n*)			
Snoring	89.1 (106)	86.4 (76)	0.67
Reported apneas	66.4 (79)	60.2 (53)	0.38
Choking	39.5 (47)	39.8 (35)	0.99
Nocturia	52.9 (63)	48.9 (43)	0.58
Awakenings	38.7 (46)	29.5 (26)	0.19
Sweating	22.7 (27)	17 (15)	0.38
Comorbidity (%, *n*)			
COPD	21 (25)	6.9 (6)	0.005
Asthma	10.1 (12)	12.6 (11)	0.66
Arterial hypertension	74.8 (89)	65.5 (57)	0.16
Chronic ischemic cardiomyopathy	10.1 (12)	11.4 (10)	0.82
Heart arrhythmias	14.3 (17)	14.8 (13)	0.99
Type 2 diabetes mellitus	22.7 (27)	29.5 (26)	0.33
Chronic kidney failure	2.5 (3)	2.3 (2)	0.99
GERD	13.4 (16)	18.2 (16)	0.44
Cancer	6.7 (8)	13.6 (12)	0.1
Thyroid diseases	25.2 (30)	23.9 (21)	0.87
Anxiety/depression	18.5 (22)	15.9 (14)	0.71
Total comorbidities (Median, IQR)	4 [2–5]	3 [2–5]	0.61

Abbreviations: OSA, obstructive sleep apnea; NRF, nocturnal respiratory failure; SD, standard deviation; IQR, interquartile range; ESS, Epworth sleepiness scale; COPD, chronic obstructive pulmonary disease; GERD, gastroesophageal reflux disease.

**Table 2:** Baseline diagnostic and treatment features according to NRF diagnosis.

	OSA with NRF (*n* = 119)	OSA Without NRF (*n* = 88)	*p* Value
HSAT (Median, IQR)			
AHI (ev/h)	53 [31.8–75.3]	25.1 [18.8–40.8]	<0.0001
ODI (ev/h)	53.9 [30.1–73.8]	23.4 [17.6–36.3]	<0.0001
TST90 (%)	50 [38.7–67]	6 [1.7–14.7]	<0.0001
TST85 (%)	9.9 [3–19]	0.2 [0–0.7]	<0.0001
TST80 (%)	2.9 [0.1–6.9]	0 [0–0.1]	<0.0001
Hypoxic burden (%min/h)	66.6 [31.1–142.3] (n = 67)	37.9 [26.9–51.1] (n = 36)	0.003
Nadir SpO_2_ (%)	70 [62–77]	80 [75–83]	<0.0001
Diagnosis (%, *n*)			
Mild OSA	6.7 (8)	12.5 (11)	0.22
Moderate OSA	16.8 (20)	45.5 (40)	<0.0001
Severe OSA	76.5 (91)	42 (37)	<0.0001
OHS	5.9 (7)	1.1 (1)	0.14
Treatment (%, *n*)			
CPAP	87.4 (104)	77.3 (67)	0.06
APAP	0.8 (1)	22.7 (20)	<0.0001
BPAP	11.8 (14)	1.1 (1)	0.003
LTOT	8.4 (10)	1.1 (1)	0.03
PAP Treatment features			
Interface (%, *n*)			
Nasal	50.4 (60)	50 (44)	0.99
Oronasal	48.7 (58)	45.5 (40)	0.67
Nasal pillow	0.8 (1)	4.5 (4)	0.17
CPAP titration pressure (cmH20, Median, IQR)	11.7 [10–13]	10 [9–11.4]	<0.0001

Abbreviations: OSA, obstructive sleep apnea; NRF, nocturnal respiratory failure; IQR, interquartile range; HSAT, home sleep apnea testing; AHI, apnea-hypopnea index; ODI, oxygen desaturation index; TST, total sleep time; SpO_2_, peripheral oxygen saturation; OHS, obesity hypoventilation syndrome; CPAP, continuous positive airway pressure; APAP, automatic positive airway pressure; BPAP, bilevel positive airway pressure; LTOT, long-term oxygen therapy; PAP, positive airway pressure.

**Table 3:** Adherence and response to treatment in patients with OSA with NRF vs OSA withot NRF.

Follow Up Visit	OSA with NRF (*n* = 119)	OSA Without NRF (*n* = 88)	*p* Value
Weight (Kg, Median, IQR)	97 [85–115]	84.5 [75–92]	<0.0001
ESS (Median, IQR)	3 [2–5]	4 [2–7]	0.01
Sleep Hours (Median, IQR)	6 [4.5–7]	6 [5.1–6.5]	0.61
PAP Treatment features			
Time to follow up visit (days, Median, IQR)	271 [118–621]	513 [313–779]	<0.0001
Reported device use (h, Median, IQR)	5.5 [4.0–6.5]	4.5 [2.1–5.5]	0.0008
Registered device use (h, Median, IQR)	5.3 [3.4–6.2]	4.0 [1.3–5.5]	0.002
Registered adherence ≥ 4.0 h (%, n)	69.7 (83)	52.3 (46)	0.01
Discomfort reasons (n, %)			
Dry nose/mouth	28.6 (34)	30.7 (27)	0.76
Interface discomfort	11.8 (14)	12.5 (11)	0.99
Air leaks	14.3 (17)	9.1 (8)	0.29
Aerophagia	0	2.3 (2)	0.18
Claustrophobia	2.5 (3)	6.8 (6)	0.17
Nasal congestion	4.2 (5)	5.7 (5)	0.75
Eye irritation/lacrimation	3.4 (4)	1.1 (1)	0.4
High titration pressure	3.4 (4)	1.1 (1)	0.4
Low titration pressure	4.2 (5)	2.3 (2)	0.7
Noise	1.7 (2)	3.4 (3)	0.65
Bed partner discomfort	1.7 (2)	0	0.51

Abbreviations: OSA, obstructive sleep apnea; NRF, nocturnal respiratory failure; IQR, interquartile range; ESS, Epworth sleepiness scale; PAP, positive airway pressure.

**Table 4:** Kaplan-Meier analysis for PAP treatment adherence according to the clinical and HSAT features of the enrolled population.

Adherence to PAP Therapy	χ^2^	*p* Value
Gender (Male)	3	0.08
Age (years)	2.3	0.31
<55		
55–64		
≥65		
Baseline ESS ≥ 10	0.03	0.86
Diagnosis	1.7	0.43
Mild OSA		
Moderate OSA		
Severe OSA		
ODI (ev/h)	2.3	0.31
0–15		
>15–30		
>30		
Nocturnal hypoxic metrics		
TST90 ≥ 30%	16.1	<0.0001
TST90 > median (34.9%)	9.4	0.002
TST85 > median (3.05%)	0.79	0.37
TST80 > median (0.1%)	1.2	0.28
Mean SpO_2_ > median (90.1%)	6.2	0.01
Nadir SpO_2_ > median (76%)	5.1	0.02
Hypoxic burden > median (45.6%min/h)	2.8	0.1

Abbreviations: PAP, positive airway pressure; ESS, Epworth sleepiness scale; OSA, obstructive sleep apnea; ODI, oxygen desaturation index; ev, events; TST, total sleep time; SpO_2_, peripheral oxygen saturation.

**Table 5:** LASSO Cox and logistic regression models for sufficient PAP therapy adherence TST90 ≥ 30% as cut-off for representing NRF. Coefficients shrunk to zero at the selected λ are reported as 0 and indicate variables not retained in the model.

	LASSO Cox Regression	LASSO Logistic Regression
Best lambda	0.04	0.02
AUC (95% CI)	0.6 (0.51–0.68)	0.66 (0.58–0.73)
*p*-values	0.02	0.006
Variables	Coefficients	
Time from PAP therapy start	0	0
Age	0	0.01
Sex	0.06	0
OSA severity	0	0
Therapy		
CPAP (Reference)	0	0
APAP	0	0
BPAP	−0.44	−1.23
Interface		
Nasal (Reference)	0	0
Oronasal	−0.51	0
Nasal pillows	−0.61	−0.07
TST90 ≥ 30%	0.58	0.31

Abbreviations: LASSO, least absolute shrinkage and selection operator; AUC, area under the curve; CI, confidence interval; PAP, positive airway pressure; OSA, obstructive sleep apnea; CPAP, continuous positive airway pressure; APAP, automatic positive airway pressure; BPAP, bilevel positive airway pressure; TST, total sleep time.

## Data Availability

The data that support the findings of this study are available from the corresponding author upon reasonable request.

## Supplementary Materials

The following supporting information can be downloaded at: https://www.mdpi.com/article/10.3390/clockssleep8040061/s1.

## Abbreviations

The following abbreviations are used in this manuscript:

AHI	Apnea-hypopnea index
APAP	Automatic positive airway pressure
AUC	Area under the curve
BMI	Body mass index
BPAP	Bilevel positive airway pressure
COPD	Chronic obstructive pulmonary disease
CPAP	Continuous positive airway pressure
ESS	Epworth sleepiness scale
HB	Hypoxic burden
HSAT	Home sleep apnea testing
IQR	Interquartile range
LASSO	Least absolute shrinkage and selection operator
NRF	Nocturnal respiratory failure
ODI	Oxygen desaturation index
OHS	Obesity hypoventilation syndrome
OSA	Obstructive sleep apnea
PAP	Positive airway pressure
REM	Rapid eye movement
ROC	Receiver operating characteristic
SD	Standard deviation
SpO_2_	Peripheral oxygen saturation
TST80	Total sleep time with SpO_2_ < 80%
TST85	Total sleep time with SpO_2_ < 85%
TST90	Total sleep time with SpO_2_ < 90%
WASO	Wakefulness time after sleep onset
