# Supplementary material for: Can Sleep Hypoxic Measures Predict Long-Term Adherence to Positive Airway Pressure Treatments?
Source: Clocks Sleep. 2026 Sep 28;8(4):61. doi: 10.3390/clockssleep8040061 (PMC13692412; doi:10.3390/clockssleep8040061)
Supplement: Supplementary file 1 [file clockssleep-08-00061-s001.zip › clockssleep-4528635-supplementary.pdf]

clocks & sleep

MDPI

Article

Can Sleep Hypoxic Measures Predict Long-Term Adherence to Positive Airway Pressure Treatments?

Andrea Portacci [1,†], Sara Quaranta [1,†], Carla Santomasi [1], Valentina Di Lecce [1], Vitaliano Nicola Quaranta [1], Monica Amendolara [1], Ilaria Dei Lazzaretti [1], Laura Anna Sara Cuccaro [1], Massimo Casparrini [1], Sebastiano Spierto [1], Vito Picerno [1], Cristina Derobertis [1], Alfredo Vozza [2], Silvano Dragonieri [1,*] and Giovanna Elisiana Carpagnano [1]

[1] Institute of Respiratory Disease, Department of Translational Biomedicine and Neuroscience, University "Aldo Moro", 70124 Bari, Italy; a.portacci01@gmail.com (A.P.); sara.quaranta.02@gmail.com (S.Q.); carlasantomasi@gmail.com (C.S.); valentina.dilecce89@gmail.com (V.D.L.); vitalianonicola.40@gmail.com (V.N.Q.); m.amendolara2@studenti.uniba.it (M.A.); ilariadeilazzaretti@gmail.com (I.D.L.); cuccarolaura@gmail.com (L.A.S.C.); maxadhar@gmail.com (M.C.); spierto.sebastiano@gmail.com (S.S.); vito.picerno.97@gmail.com (V.P.); cri.derobertis@gmail.com (C.D.); elisiana.carpagnano@uniba.it (G.E.C.)

[2] Department of Precision and Regenerative Medicine and Ionian Area, University of Bari "Aldo Moro", 70124 Bari, Italy; alfredovozza@live.it

* Correspondence: silvano.dragonieri@uniba.it

† These authors contributed equally to this work.

 https://doi.org/10.3390/clockssleep8040061

S1. Supplemental Material

Table S1. Symptoms at follow up visit in patients with OSA with NRF vs OSA without NRF.

	**Worsened**		**Unchanged**		**Improved**		**Absent**		**P value**
	**OSA with NRF**	**OSA without NRF**	**OSA with NRF**	**OSA without NRF**	**OSA with NRF**	**OSA without NRF**	**OSA with NRF**	**OSA without NRF**	
**Daytime Symptoms (%, n)**									
Sleepiness	5 (6)	6.8 (6)	12.6 (15)	30.7 (27)	24.4 (29)	21.6 (19)	58 (69)	40.9 (36)	**0.009**
Fatigue	9.2 (11)	6.8 (6)	26.9 (32)	36.4 (32)	16.8 (20)	11.4 (10)	47.1 (56)	45.5 (40)	0.41
Headache	2.5 (3)	3.4 (3)	9.2 (11)	18.2 (16)	8.4 (10)	2.3 (2)	79.8 (95)	76.1 (67)	0.09
Insomnia	5.9 (7)	8 (7)	16.8 (20)	35.2 (31)	18.5 (22)	14.8 (13)	58.8 (70)	42 (37)	**0.01**
Memory Loss	6.7 (8)	6.8 (6)	24.4 (29)	40.9 (36)	6.7 (8)	3.4 (3)	62.2 (74)	48.9 (43)	0.07
Concentration disorders	4.2 (5)	5.7 (5)	19.3 (23)	38.6 (34)	5.9 (7)	4.5 (4)	70.6 (84)	51.1 (45)	**0.02**
Mood disorders	3.4 (4)	4.5 (4)	30.3 (36)	34.1 (30)	4.2 (5)	0	62.2 (74)	61.4 (54)	0.25
**Nighttime symptoms (%, n)**									
Snoring	1.7 (2)	0	5 (6)	23.9 (21)	11.8 (14)	15.9 (14)	81.5 (97)	60.2 (53)	**< 0.0001**
Reported Apneas	1.7 (2)	1.1 (1)	6.7 (8)	14.8 (13)	7.6 (9)	5.7 (5)	84 (100)	78.4 (69)	0.28
Choking	3.4 (4)	1.1 (1)	8.4 (10)	15.9 (14)	6.7 (8)	3.4 (3)	81.5 (97)	79.5 (70)	0.21
Nocturia	3.4 (4)	1.1 (1)	22.7 (27)	40.9 (36)	11.8 (14)	4.5 (4)	62.2 (74)	53.4 (47)	**0.02**
Awakenings	5 (6)	3.4 (3)	19.3 (23)	26.1 (23)	6.7 (8)	4.5 (4)	68.9 (82)	65.9 (58)	0.6
Sweating	1.7 (2)	4.5 (4)	12.6 (15)	15.9 (14)	3.4 (4)	0	82.4 (98)	79.5 (70)	0.18

Abbreviations: OSA, obstructive sleep apnea; NRF, nocturnal respiratory failure.

Table S2. LASSO Cox and logistic regression models for sufficient PAP therapy adherence using the TST90 > its median value as cut-off for the analysis.

	LASSO Cox regression	LASSO logistic regression
**Best lambda**	0.04	0.03
**AUC (95% CI)**	0.59 (0.5-0.67)	0.66 (0.58-0.74)
**P-values**	0.02	< 0.0001
**Variables**	**Coefficients**	
**Time from PAP therapy start**	0	0
**Age**	0	0.01
**Sex**	0.06	0
**OSA severity**	0	0
**Therapy**		
CPAP (Reference)	0	0
APAP	0	0
BPAP	-0.57	-1.34
**Interface**		
Nasal (Reference)	0	0
Oronasal	-0.46	0
Nasal pillows	-0.67	-0.11
**TST90 > median**	0.35	0.05

Coefficients shrunk to zero at the selected λ are reported as 0 and indicate variables not retained in the model.

Abbreviations: LASSO, least absolute shrinkage and selection operator; AUC, area under the curve; CI, confidence interval; PAP, positive airway pressure; OSA, obstructive sleep apnea; CPAP, continuous positive airway pressure; APAP, automatic positive airway pressure; BPAP, bilevel positive airway pressure; TST, total sleep time.

Table S3. LASSO Cox and logistic regression models for sufficient PAP therapy adherence using the lowest SpO2 > its median value as cut-off for the analysis.

	LASSO Cox regression	LASSO logistic regression
**Best lambda**	0.04	0.02
**AUC (95% CI)**	0.61 (0.53-0.7)	0.66 (0.59-0.74)
**P-values**	0.03	< 0.0001
**Variables**	**Coefficients**	
**Time from PAP therapy start**	0	0
**Age**	0.003	0.02
**Sex**	0	0
**OSA severity**	0	0
**Therapy**		
CPAP (Reference)	0	0
APAP	0	0
BPAP	-0.67	-1.38
**Interface**		
Nasal (Reference)	0	0
Oronasal	-0.55	0
Nasal pillows	-0.78	-0.16
**Lowest SpO2 > median**	-0.34	-0.18

Coefficients shrunk to zero at the selected λ are reported as 0 and indicate variables not retained in the model.

Abbreviations: LASSO, least absolute shrinkage and selection operator; AUC, area under the curve; CI, confidence interval; PAP, positive airway pressure; OSA, obstructive sleep apnea; CPAP, continuous positive airway pressure; APAP, automatic positive airway pressure; BPAP, bilevel positive airway pressure; TST, total sleep time.

https://doi.org/10.3390/clockssleep8040061

Table S4. LASSO Cox and logistic regression models for sufficient PAP therapy adherence using the mean SpO2 > its median value as cut-off for the analysis.

	LASSO Cox regression	LASSO logistic regression
**Best lambda**	0.04	0.03
**AUC (95% CI)**	0.58 (0.49-0.67)	0.66 (0.58-0.74)
**P-values**	0.03	0.001
**Variables**	**Coefficients**	
**Time from PAP therapy start**	0	0
**Age**	0	0.01
**Sex**	0.05	0
**OSA severity**	0	0
**Therapy**		
CPAP (Reference)	0	0
APAP	0	0
BPAP	-0.72	-1.37
**Interface**		
Nasal (Reference)	0	0
Oronasal	-0.49	0
Nasal pillows	-0.72	-0.12
**Mean SpO2 > median**	-0.31	0

Coefficients shrunk to zero at the selected λ are reported as 0 and indicate variables not retained in the model.

Abbreviations: LASSO, least absolute shrinkage and selection operator; AUC, area under the curve; CI, confidence interval; PAP, positive airway pressure; OSA, obstructive sleep apnea; CPAP, continuous positive airway pressure; APAP, automatic positive airway pressure; BPAP, bilevel positive airway pressure; TST, total sleep time.

Table S5. Multivariable logistic regression analysis of nocturnal hypoxia metrics associated with good PAP adherence.

**Predictor**	**Adjusted OR (95% CI)**	**P value**	**AUC**
NRF	2.52 (1.24-5.13)	**0.01**	0.65
TST90 (per 10% -point increase)	1.12 (0.97-1.29)	0.12	0.62
Hypoxic burden > median	0.46 (0.15-1.41)	0.17	0.69
TST90 > median	1.85 (0.93-3.68)	0.08	0.62
TST85 > median	1.13 (0.36-3.54)	0.83	0.67
TST80 > median	0.80 (0.27-2.37)	0.69	0.67
Mean SpO2 > median	0.76 (0.4-1.44)	0.40	0.59
Lowest SpO2 > median	0.54 (0.27-1.07)	0.08	0.62

Models were adjusted for age, sex, BMI, AHI, COPD, total number of comorbidities, follow-up duration, type of treatment (CPAP/APAP vs BPAP), LTOT and interface type (nasal/nasal pillows vs oronasal).

Abbreviations: OR, odds ratio; CI, confidence interval; AUC, area under the curve; NRF, nocturnal respiratory failure; TST, total sleep time; SpO2, peripheral oxygen saturation.

https://doi.org/10.3390/clockssleep8040061

Figure S1. Schematic illustration of hypoxic burden calculation. (A) The area of each desaturation is calculated between the baseline SpO_2 and the desaturation curve within the interval defined by the pre-maximum and post-maximum. (B) The areas of retained, non-overlapping desaturations are summed and divided by the normalization time to obtain hypoxic burden. This figure was created with the assistance of generative artificial intelligence (ChatGPT, OpenAI GPT-5.6 Sol model).

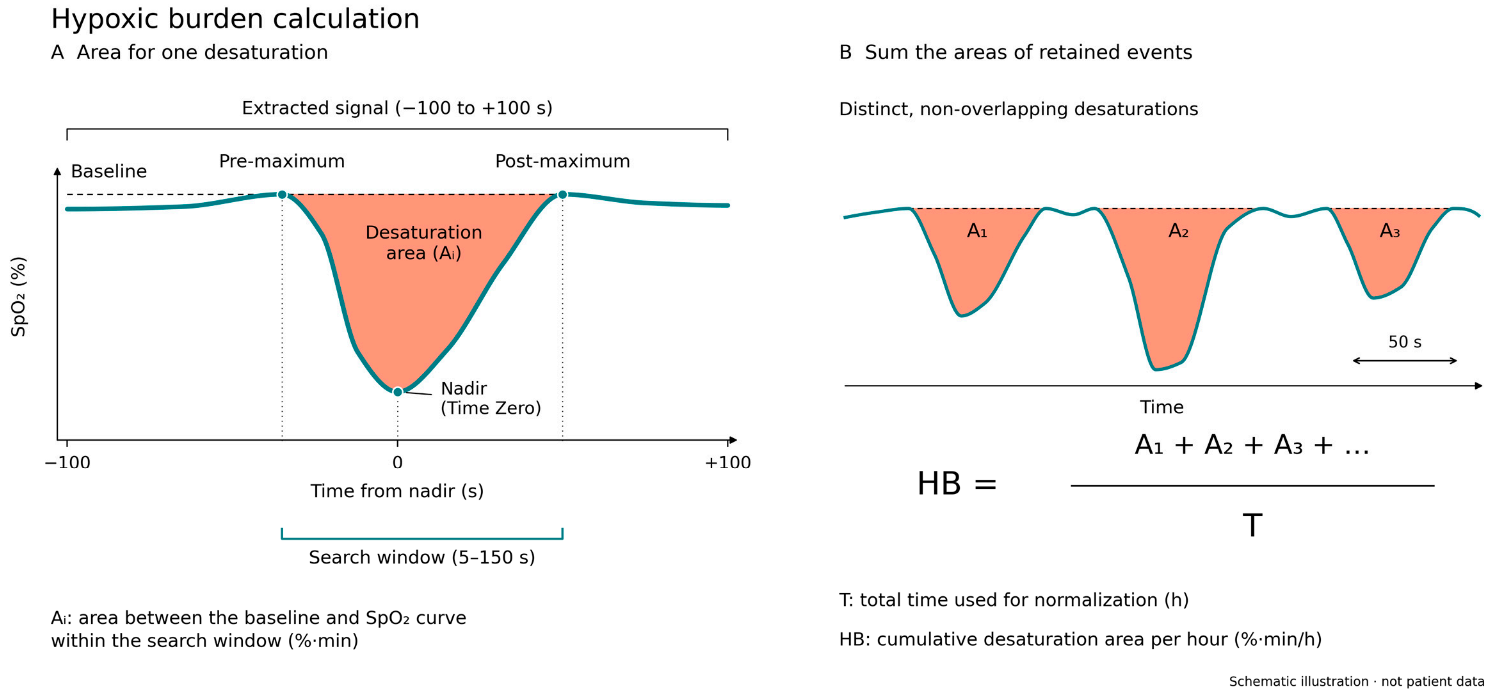

Abbreviations: HB, hypoxic burden (%·min/h); SpO_2, peripheral oxygen saturation; A_i, area of an individual desaturation (%·min); T, total time used for normalization (h); s, seconds; min, minutes; h, hours.
